# Literature mining discerns latent disease–gene relationships

**DOI:** 10.1093/bioinformatics/btae185

**Published:** 2024-04-12

**Authors:** Priyadarshini Rai, Atishay Jain, Shivani Kumar, Divya Sharma, Neha Jha, Smriti Chawla, Abhijit Raj, Apoorva Gupta, Sarita Poonia, Angshul Majumdar, Tanmoy Chakraborty, Gaurav Ahuja, Debarka Sengupta

**Affiliations:** Department of Computational Biology, Indraprastha Institute of Information Technology-Delhi (IIIT-Delhi), Okhla Phase III, New Delhi 110020, India; Department of Computer Science and Engineering, Indraprastha Institute of Information Technology-Delhi (IIIT-Delhi), Okhla Phase III, New Delhi 110020, India; Department of Computer Science and Engineering, Indraprastha Institute of Information Technology-Delhi (IIIT-Delhi), Okhla Phase III, New Delhi 110020, India; Department of Computational Biology, Indraprastha Institute of Information Technology-Delhi (IIIT-Delhi), Okhla Phase III, New Delhi 110020, India; Department of Computational Biology, Indraprastha Institute of Information Technology-Delhi (IIIT-Delhi), Okhla Phase III, New Delhi 110020, India; Department of Computational Biology, Indraprastha Institute of Information Technology-Delhi (IIIT-Delhi), Okhla Phase III, New Delhi 110020, India; Department of Computational Biology, Indraprastha Institute of Information Technology-Delhi (IIIT-Delhi), Okhla Phase III, New Delhi 110020, India; Department of Biotechnology, Delhi Technological University, Shahbad Daulatpur, Delhi 110042, India; Department of Computational Biology, Indraprastha Institute of Information Technology-Delhi (IIIT-Delhi), Okhla Phase III, New Delhi 110020, India; IAI, TCG CREST, Kolkata 700091, India; Department of Electrical Engineering, Indian Institute of Technology Delhi, New Delhi 110016, India; Yardi School of Artificial Intelligence, Indian Institute of Technology Delhi, New Delhi 110016, India; Department of Computational Biology, Indraprastha Institute of Information Technology-Delhi (IIIT-Delhi), Okhla Phase III, New Delhi 110020, India; Centre for Artificial Intelligence, Indraprastha Institute of Information Technology-Delhi (IIIT-Delhi), Okhla Phase III, New Delhi 110020, India; Department of Computational Biology, Indraprastha Institute of Information Technology-Delhi (IIIT-Delhi), Okhla Phase III, New Delhi 110020, India; Department of Computer Science and Engineering, Indraprastha Institute of Information Technology-Delhi (IIIT-Delhi), Okhla Phase III, New Delhi 110020, India; Centre for Artificial Intelligence, Indraprastha Institute of Information Technology-Delhi (IIIT-Delhi), Okhla Phase III, New Delhi 110020, India

## Abstract

**Motivation:**

Dysregulation of a gene’s function, either due to mutations or impairments in regulatory networks, often triggers pathological states in the affected tissue. Comprehensive mapping of these apparent gene–pathology relationships is an ever-daunting task, primarily due to genetic pleiotropy and lack of suitable computational approaches. With the advent of high throughput genomics platforms and community scale initiatives such as the Human Cell Landscape project, researchers have been able to create gene expression portraits of healthy tissues resolved at the level of single cells. However, a similar wealth of knowledge is currently not at our finger-tip when it comes to diseases. This is because the genetic manifestation of a disease is often quite diverse and is confounded by several clinical and demographic covariates.

**Results:**

To circumvent this, we mined ∼18 million PubMed abstracts published till May 2019 and automatically selected ∼4.5 million of them that describe roles of particular genes in disease pathogenesis. Further, we fine-tuned the pretrained bidirectional encoder representations from transformers (BERT) for language modeling from the domain of natural language processing to learn vector representation of entities such as genes, diseases, tissues, cell-types, etc., in a way such that their relationship is preserved in a vector space. The repurposed BERT predicted disease–gene associations that are not cited in the training data, thereby highlighting the feasibility of *in silico* synthesis of hypotheses linking different biological entities such as genes and conditions.

**Availability and implementation:**

PathoBERT pretrained model: https://github.com/Priyadarshini-Rai/Pathomap-Model. BioSentVec-based abstract classification model: https://github.com/Priyadarshini-Rai/Pathomap-Model. Pathomap R package: https://github.com/Priyadarshini-Rai/Pathomap.

## 1 Introduction

The number of published biomedical manuscripts has sharply increased since the beginning of the genomic era. Nearly 3000 manuscripts are published daily in peer-reviewed journals (Lee *et al.* 2019). This has resulted in a voluminous corpus of scientific literature, outreaching by far, the limit of human comprehension. Given the complexity of human biological systems, it is nearly impossible to get a qualitative as well as a quantitative view of organ/tissue wide pathological roles of genes by an internet search of the literature. Today, a great deal of community level effort is channeled towards creating a molecular atlas of healthy human organs by charting out the functionally distinct cellular subtypes in tissues of interest. Such effort is scarce in disease biology due to the presence of innumerable covariates and the diversity of the disease states. A vast majority of knowledge-based archiving disease–gene association is painstakingly, manually curated. DisGeNET (Piñero *et al.* 2017) and Online Mendelian Inheritance in Man (OMIM^®^) (McKusick 2007) are noteworthy in this regard. Natural language processing (NLP)-based efforts in this regard have mainly concentrated on extraction of disease–gene association from biomedical literature which is an automated version of manual data curation (Bravo et al. 2015). Quan and Ren (2014) used Probabilistic Context–Free Grammars to extract disease–gene associations (), whereas Zhou and Fu (2018) used a simple, term co-occurrence based method for the same task.

Recent invention of distributed representation words has transformed the field of text mining. Continuous embedding of words typically uses efficient neural networks to learn word-representations by ingesting millions of documents. Mikolov *et al.* (2013) first proposed the very idea of learning a continuous bag of words representation of words at Google LLC. The field has since evolved dramatically, and in recent times, transformer-based models such as BERT (bidirectional encoder representations from transformers) (Devlin *et al.* 2018) have emerged as state-of-the-art. In the past years, text mining and language modeling have gained prominence in the fields of biomedical research. Gideon *et al.* used word embedding techniques to identify associations among different brain regions (Rosenthal *et al.* 2018). Similar approaches have been used in predicting compound–protein interactions (Wan and Zeng 2016) and drug discovery (Agarwal and Searls 2008). BERT is highly resource intensive. In a recent effort by Lee and colleagues, embeddings have been produced for several billions of words by processing voluminous biomedical literature, using BERT. We hypothesized that such generic word embeddings may not capture the essence of molecular pathology. To circumvent this, we developed a systematic strategy to identify abstracts that describe association between certain genes’ dysregulation and diseases from a database of ∼18 million biomedical abstracts (Kim *et al.* 2019). About 4.5 million selected abstracts were then used for fine-tuning the BERT base language model (referred to as *PathoBERT*), while following NLP best practices. We found few studies that utilized word embedding techniques to extract sentences featuring disease–gene relationships (Kim *et al.* 2013). These tools are, by default, not capable of extracting latent knowledge regarding disease–gene associations that might not be directly captured in publications. Notably, this area also features a different school of approaches that involve node embedding in heterogeneous networks spanning genes, diseases, chemicals, etc. Notable among these is the work by Zhou *et al.* (2022), which provides ∼11.2 two million putative disease–gene connections. A similar work by Yang *et al.* (2019) used disease-related (e.g. symptoms) and gene-related (e.g. gene ontology and protein–protein interactions) information to improve the performance of disease gene prediction. Yang *et al.*’s (2020) survey paper on representation learning on heterogeneous networks is a great resource in this regard. Network-based approaches are extremely useful but contingent on availability of comprehensive network topologies. Furthermore, these methods have not been demonstrated to be useful for representing latent knowledge or churning out new discoveries.

We examined *PathoBERT*’s ability to represent genes, diseases, organs/tissues, cell-types and their inter-relationships in the associated embedding space. We benchmarked *PathoBERT* and other embedding techniques against gold-standard manually curated disease–gene relationships. *PathoBERT* by far exceeded the performance of the existing ready-to-use embeddings. We also developed an R package, *Pathomap*, to enable visualization of genes’ tissue-specific pathological roles as a heatmap on a human body layout. Furthermore, these disease-focused word embeddings allowed us to extract latent knowledge beyond the ambit of the training literature corpus.

## 2 Materials and methods

### 2.1 Data description

We downloaded ∼18 million abstracts (Kim *et al.* 2019) from PubMed that were published till May 2019. We postulated that abstracts capture the essence of the entire manuscript (Tshitoyan *et al.* 2019). Also, due to paywalls, many articles are not accessible for mining.

### 2.2 Manual annotation of abstracts

To train a model that automatically classifies disease–gene related abstracts (*patho-abstracts*) versus unrelated ones, we first manually curated a limited number of abstracts that constitute positive and negative samples respectively. For this, we looked for articles in the field of molecular biology and genetics. We primarily referred to articles from The NHGRI-EBI GWAS Catalog (Buniello *et al.* 2019), COSMIC (Catalog Of Somatic Mutations In Cancer) (Bamford *et al.* 2004), and OMIM (Online Mendelian Inheritance in Man) databases. This led to creation of two sets of abstracts—first, describing genes’ direct roles in disease pathogenesis, and second, abstracts that do not cite apparent disease–gene relationships. The abstracts with information about gene–disease relationships were labeled as “relevant” or “positive” data and the remaining abstracts that did not mention disease–gene connections were labeled as “nonrelevant” or “negative” data. The cohort of pathological abstracts consists of 1412 abstracts and the group of nonpathological abstracts consists of 687 abstracts (Supplementary File S1). Each abstract was annotated by a group of three annotators (co-authors in this paper) as “relevant” or “nonrelevant.” Then we computed the alpha-reliability inter-annotator agreement (Krippendorff 2011) between each pair of annotators, αAB = 0.83, αAC = 0.84, αBC = 0.99. Final annotations were obtained using the majority voting procedure.

### 2.3 Stratifying pathological abstracts from nonpathological abstracts

An abstract stratification strategy was developed to shortlist the abstracts that depict the pathological role of genes. We used positive and negative sets of abstracts as seed data. These abstracts were further divided into train and test sets in a 75:25 split. We performed a basic set of preprocessing steps for each abstract that comprises lowercasing the whole text and removing stop words and punctuations. Further, in order to obtain a vector representation for each abstract, we use three methods:

- SkipGram: We used the basic skip-gram architecture of Word2vec to train the model for 1000 epochs to learn a 700D representation for each word present in the vocab. The window size is kept as 5 here. To get the representation for each abstract, we take the mean of all the word vectors present in the document.
- BioSentVec: We used the pre-trained model here without any finetuning since the model is already fine-tuned for biological text. We passed each abstract in natural language to the model and the model returned a 700D vector (Chen *et al.* 2019).
- BioBERT: It is a BERT-based model which is fine tuned on biological data. It gives a 768D representation for each subword. Finally, we considered the mean of these embeddings to get the representation for the complete abstract.

After we obtained the representation for the abstracts by all of the described methods, we moved on to train ML-based classification models for classifying an unseen abstract into pathological versus nonpathological subtypes. We used the Sklearn library (Marcelino *et al.* 2017) for all the classifiers. We performed a grid search to obtain optimal hyperparameters and report the results in Supplementary Table S1. Below are descriptions of the methods, cross-compared on the abstract classification tasks.

- Support vector machine (SVM): We fitted a three-degree polynomial kernel with balanced class weights to learn a mapping between the representation and the binary labels that determines whether the abstract is pathological or not (ChangChih-Chung and Lin 2011).
- Extreme gradient boosting (XGBoost): We used 100 estimators with 40 jobs and a learning rate of 3e−1 to learn the required mapping (Sharma 2018).
- Logistic regression (LR): The default L2 regularization with a lbfgs solver converged in around 100 iterations to give us the required mapping (Hosmer *et al.* 2013).
- Random Forest: With a maximum depth of 2, we learned the random forest architecture (Ho 2002).
- Multi-layer perceptron (MLP): We learned a simple multi-layer perceptron within 300 iterations (Gardner and Dorling 1998).

The XGBoost model provided overall best performance in comparison to other classifiers on the majority metrics (accuracy, recall, and kappa score). We relied on *kappa* as a guiding metric due to data imbalance. Therefore, we finally used the XGBoost model learnt using the embeddings obtained from the BioSentVec model for classification of ∼18 million abstracts. As a stringent selection criterion, we set the threshold as 0.8 and obtained 4 576 952 pathological abstracts.

### 2.4 Fine-tuned PathoBERT

We aimed to extract a representation of all the ∼4.5 million pathological abstracts obtained after classification. For this, we first performed tokenization using Python's NLTK's word tokenizer (Perkins 2010). After that, we removed punctuation and stop-words from the list of tokens. We found that lower-casing the articles helped reduce the computational overhead and the vocabulary size. To ensure meaning embeddings for biological concepts we used named entities (NEs) from Kim *et al.* (Kim *et al.* 2019) for all ∼18 million abstracts. Kim *et al.* used Pubtator as a backbone for NER (Wei *et al.* 2019). To this, we also included disease names as NEs from the DisGeNET web server (Piñero *et al.* 2017). The spaces in n-grams associated with the NEs were replaced by underscores (“_”). For instance, the bigram “adipose tissue” was converted to “adipose_tissue.”

For getting the vectors for these pre-processed abstracts, we explored four different methods—BioBERT (Lee *et al.* 2019), BioSentVec (Chen *et al.* 2019), BERT (Devlin *et al.* 2018), and the skip-gram variant of Word2vec (Mikolov *et al.* 2013). BioBERT and BioSentVec were used off-the-shelf. We fine-tuned the BERT-base for the masked language modeling task (source: huggingface repository) for two-epochs with our pre-processed pathological abstracts. To get the representations associated with a query (word/phrase/sentence/paragraphs), we used mean representation from the last four layers of the fine-tuned BERT model (called *Patho-BERT*). We used a 32 GB Tesla V100 GPU for this. A skip-gram model was trained from scratch using the pathological abstracts to obtain an embedding for a query word. Only ∼1.2 million of the ∼4.5 million abstracts were tagged with species that could be relatable to humans (human, boy, girl, children, man, woman, men, women, patients, and patient). Based on these further filtered abstracts we created fine-tuned versions of *Patho-BERT* and *skip-gram*.

### 2.5 Word similarity measurement

To measure semantic similarity between a pair of words we used the cosine distance between the associated word vectors. Cosine distance in this case is called *Patho-score* since it measures the extent to which a pair of words occurs together in pathology related publications.

### 2.6 Pathomap for visualizing the impact of a gene on different tissues

We created an R package that allows users to visualize a gene’s tissue specific pathogenic roles and compare them with tissue-wide expression levels, obtained from GTEx gene expression archives (Lonsdale *et al.* 2013). We used the *gganatogram* R package to visualize *Patho-scores* on a human body template (Maag 2018). For visualizing gene expression levels, we applied median normalization and log transformation to the GTEx expression matrix.

### 2.7 Monte-Carlo estimation of *P*-value associated with Patho-scores

Word-embedding tools are unsupervised and therefore not free from noise and spurious embeddings. As such, analysis of statistical significance is crucial. We computed *P*-value

(1) $$P=\frac{\#\left(PS\;>\;r_{i}\right)\;\forall \;i\;+\;1}{\left|r\right|\;+\;1}$$

where *PS* is the observed *Patho-score*, and *r* is the set of *Patho-scores* computed between random word pairs (Tissue-Gene pairs or Disease–Gene pairs depending on the context).

### 2.8 Network diffusion-based novel marker discovery in colorectal cancer

We used a genome-wide multiplex network composed of three connection types—(i) disease–disease, (ii) disease–gene, and (iii) protein–protein (https://academic.oup.com/bioinformatics/article/35/3/497/5055408). Random Walk with Restart (RWR) on such a multiplex network can be defined as follows.

(2) $$p_{t+1}=\left(1-r\right)Mp_{t}+rp_{0}$$

where *M* is a degree normalized adjacency matrix, and the vector *p_0_* is the initial probability distribution, with seed nodes having nonzero values. After several iterations, the difference between the vectors *p_t+1_* and *p_t_* becomes negligible, and the stationary probability distribution is reached. Elements in these vectors represent a proximity measure from every graph node to the seed(s). Restart probability *r* is set to 0.7. A solution to an RWR is attained through eigen-decomposition or numerical methods such as the power iteration algorithm. We used the R package *RandomWalkRestartMH* to perform RWR with “colorectal cancer” and “*PLA2G2A*” (known to be associated with the disease) as seeds. We assessed some rather unknown proteins in the neighborhood of the seed nodes with help of *PathoBERT*.

## 3 Results and discussion

### 3.1 Training a disease-focused language model

**Figure 1.**
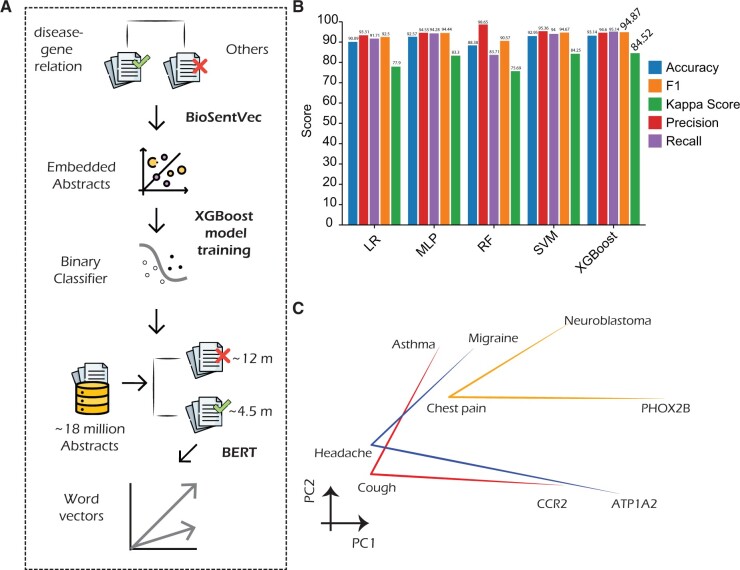
(A) Steps involved in representation learning from abstracts describing disease–gene associations. (B) BioSentVec provided based accuracy in identifying articles describing gene–disease relationships. (C) Congruence of disease–symptom–causative gene triplets in the word-vector space.

As stated in Section 1, there are numerous methods to extract disease–gene relationships by literature mining. While these models do a decent job in automatically identifying literature evidence for disease–gene association, these are not trained to perform the masked-language-modeling task in the context of pathologies. As such, these are not useful for capturing the latent knowledge space spanning different biological entities in relation to diseases. To circumvent this, we classified ∼18 million abstracts into ones that describe disease–gene associations and ones that do not. This yielded ∼4.5 million *patho-abstracts.* We trained skip-gram from scratch and fine-tuned the base BERT (including versions focused entirely on literature tagged with “human” as the concerned species) with these filtered abstracts. The process flow is depicted in Fig. 1A. BioSentVec-based embeddings provided best accuracy in classifying unseen patho-abstracts from rest (Fig. 1B).

Fine-tuned BERT, termed as *Patho-BERT, provided* best outputs when benchmarked against several ground-truth concepts (described in following sections). To evaluate the meaningfulness of the word vectors, we constructed a case-study to visualize the semantic similarity between disease-symptom-gene relationships. We considered three such triplets—neuroblastoma–chest pain–*PHOX2B* (Trochet *et al.* 2005); asthma–cough–*CCR2* (Hartl *et al.* 2005); migraine–headache–*ATP1A2 (**De Fusco et al.* 2003). We visualized vectors associated with these words using Principal Component Analysis (PCA) (Fig. 1C). The relative positioning of the words across the triplets exhibits consistent directionalities, such that there exist consistent vector operations between words that represent concepts such as “symptom of” and “causative gene for.”

### 3.2 Disease linked genes are uniformly distributed across the human genome

We speculated that there could be genomic hotspots of disease linked genes. To test this, we divided the length of each of the human chromosomes into contiguous chunks spanning 1 million base pairs and counted the total number of genes located in each of these DNA stretches. Further, we counted the number of times gene(s) located in each of these stretches appeared in the *patho-abstracts*. We found these values to be highly correlated across the 1 million base-pair long DNA stretches (Supplementary Fig. S1) with a Spearman’s correlation coefficient of 0.7.

### 3.3 PathoBERT maximizes predictability of disease–gene associations

**Figure 2.**
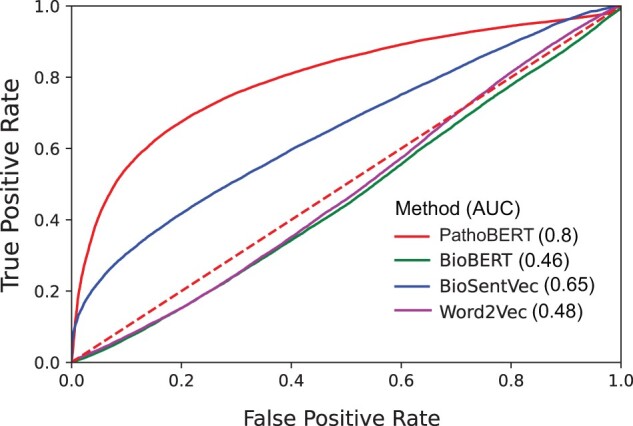
ROC plots and associated AUC values depicting the accuracy yielded using different embeddings in recognizing true gene–disease associations as compared to randomly generated ones. For this assessment, identifying disease–gene association is posed as a bi-class classification problem wherein positive associations are those which are archived in manual curation database DisGeNET, whereas negatives are created by randomly pairing genes with diseases. Note that it is hard to guarantee absence of biologically relevant association for the random cases.

As indicated in Section 2, we compared six independent models to evaluate their ability to identify manually curated ground-truth disease–gene relationships obtained from DisGeNET (Piñero *et al.* 2017). For this we constructed a positive set (associated gene–disease pairs) (Supplementary File S2) consisting of ∼36 000 disease–gene relationships (tokens for some pairs were not found in Word2vec-based embeddings), and an equivalent negative set consisting of random disease–gene pairs (Supplementary File S3). We measured cosine distance between disease–gene pairs using embeddings from different methods. ROC–AUC analysis was performed by posing detection of disease–gene association as a binary classification problem with cosine distance as the single explanatory variable. *PathoBERT* and BioSentVec showed stable predictability, with PathoBERT leading with an AUC of 0.8 (Fig. 2). BioBERT and fine-tuned Word2vec produced nonrandom accuracy. BERT and Word2vec trained on human related literature performed equally badly. Apart from superior performance, PathoBERT, due to its underlying architecture churns embeddings for any word or phrase, even if it is not present in the training corpus. We also compared our method with the DISEASES database which uses text mining for inferring gene–diseases associations (Pletscher-Frankild *et al.* 2015). The number of common gene–disease pairs between DisGeNET and DISEASES database were 13 740 whereas *Patho-BERT* had 35 467 DisGeNET gene–disease pairs whose *Patho-score*s were significant, i.e. *P*-value < 0.05 (Supplementary Fig. S2).

Apart from the embedding-based methods, we also compared PathoBERT with the two network-based methods, random walk, and DIAMONnD (Ata *et al.* 2021). Given a disease as a seed, the network-based methods provide a ranked list of all genes present in the biological network, related to the given disease. To compare the embedding-based approach, PathoBERT, with the network-based methods, we used ∼900 gene–disease pairs (Wikipedia, n.d.) to implement ROC–AUC analysis. Using PathoBERT, the AUC value for this analysis was 0.779, and using random walk (Valdeolivas *et al.* 2019) and DIAMONnd (Ghiassian *et al.* 2015), it was 0.554 and 0.540, respectively (Supplementary Fig. S3).

### 3.4 Gene’s pathological role at the resolution of organs and cell-types

**Figure 3.**
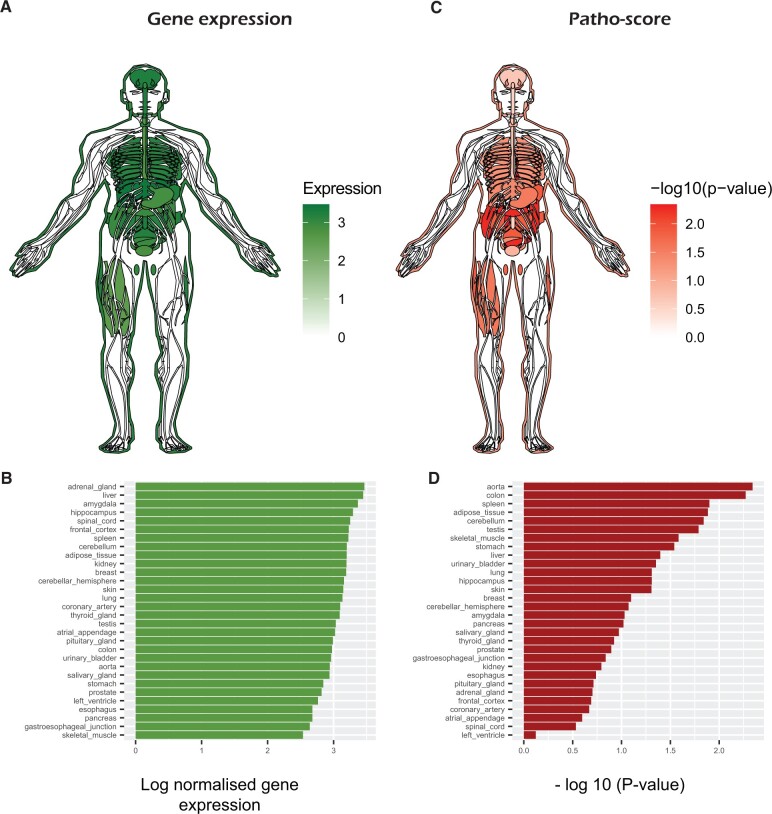
(A, B) *APOE* expression distribution across different organs. (C, D) Figures depict the pathological role of *APOE* across different organs. Statistical significance associated with *Patho-scores* are discordantly distributed across organs/tissues.

Since the beginning of the genomic era, several studies have been conducted to create molecular portraits of healthy tissues using transcriptomic, proteomic, and epigenomic assays. Single cell transcriptomics has fueled such efforts by providing unprecedented resolution into phenotypic heterogeneity in cells. With the advent of a high throughput single-cell transcriptomics platform, the past few years have seen a sharp spike in single cell atlasing effort, disentangling versatile molecular architecture of healthy tissues. It is difficult to undertake such studies for diseased tissues due to the diverse nature of genetic aberrations that underpin their associated pathogenesis. We developed an R package *Pathomap* that provides a meta-level view of gene’s pathological roles across organs as a heatmap laid on the human body template. It also provides empirically estimated statistical significance for each *Patho-score*. Figure 3 depicts organ-wide distribution of gene expression and *Patho-score* for *APOE*. APOE is linked with an increased risk of heart disease (Mahley 2016). APOE genotype might be associated with colon cancer risk and prognosis in a gender-specific manner (Watson *et al.* 2003). It is also involved in regulating lipid homeostasis. Further, the e4 version of the APOE gene increases an individual's risk of late-onset Alzheimer's disease (Liu *et al.* 2013). Alzheimer’s disease (AD) is characterized by progressive loss of memory and other cognitive abilities, as well as brain atrophy and the formation of amyloid plaques (Liu *et al.* 2013, Karch *et al.* 2014). A few more such associations are furnished in the Supplementary Material (Supplementary Figs S4–S10).

**Figure 4.**
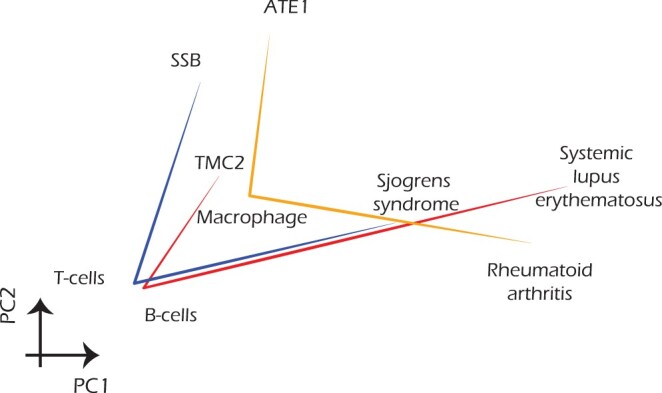
Word vectors associated with three triplets of an auto-immune disease, the impacted cell type and a well-known gene involved in disease pathogenesis.

We asked if, in contrast to organ specific pathological activity measurement, *PathoBERT* could be used to decipher cell-type specific pathological roles of a gene. To this end we created three gene–cell type–disease triplets, concerning three autoimmune diseases namely Sjogren's syndrome, Systemic lupus erythematosus, and Rheumatoid arthritis along with the impacted cell types and genes implicated in their pathogenesis. Some immune cells such as T cells, B cells, and Macrophages are known to be involved in the pathogenesis of Sjogren's syndrome (Singh and Cohen 2012), Systemic lupus erythematosus (Chan *et al.* 2013), and Rheumatoid arthritis (Yap *et al.* 2018) respectively. The information on genes involved in these diseases was sourced from the Enrichr database (Kuleshov *et al.* 2016). We created three triplets—SSB–T cells–Sjogren's syndrome; TMC2–B cells–Systemic lupus erythematosus; ATE1–Macrophage–Rheumatoid arthritis. We visualized these relationships in the PCA space utilizing the associated *PathoBERT* vectors (Fig. 4). We observed consistency in directionalities in terms of the relative positioning of the words across three triplets.

### 3.5 PathoBERT identifies disease–gene associations not directly referenced in literature

**Figure 5.**
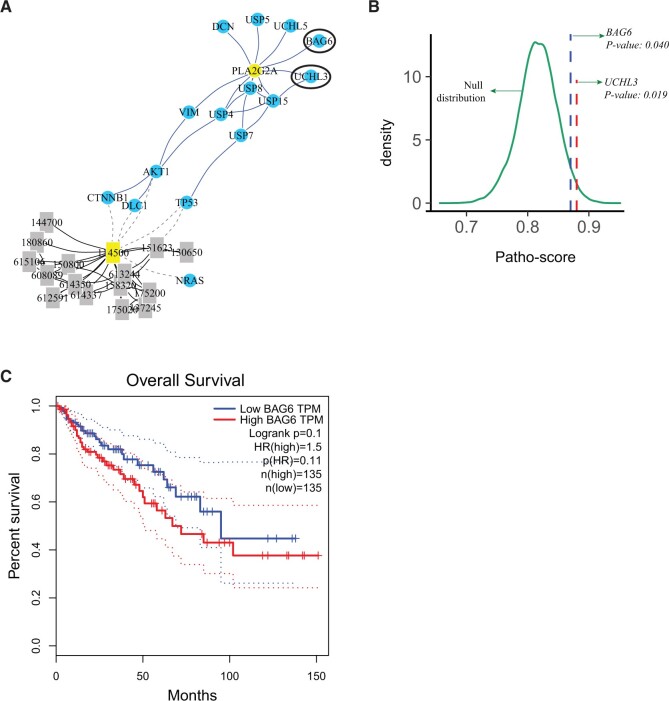
(A) Random walk with restart (RWR) in a genome wide network comprising proteins and diseases and nodes and three types of connection—(i) disease–disease similarity, (ii) protein–protein interactions, and (iii) disease–gene relationships. RWR needs seed nodes to start, which are set to be ‘colorectal cancer’ (OMIM: 114500) and *PLA2G2A* that is known to be associated with colorectal cancer. Top 15 genes and disease OMIM ids are shown on the figure. Most genes are well known for their association with colorectal cancer, except for *UCHL3* and *BAG6*. (B) Empirically calculated *P-*values calculated relative to null distribution through permutation test. (C) Overall survival estimated based on *BAG6* expression level in TCGA colon adenocarcinoma samples [generated using the GEPIA web server (Tang *et al.* 2017)].

We expected PathoBERT to capture latent knowledge inscribed in pathology related literature. Therefore, to demonstrate how PathoBERT can be a companion for discovery of new biomarkers associated with diseases, we applied Random Walk with Restart (RWR)-based network diffusion algorithm on a multiplex disease–gene network (see Section 2). As set “colorectal cancer’ and “*PLA2G2A*” (known to be associated with the disease) as seeds for this analysis. RWR provides a probability distribution for all diseases and genes, participating in the network with higher weightage to the neighborhood of the seeds. Figure 5A portrays top 15 genes and 15 disease OMIM ids, with relatively large probabilities indicating frequent accessibilities to the seeds. While most genes in the network are well-known for their implication in colorectal cancers, we found two candidates with little or no literature evidence regarding their direct implication in the disease. These are *UCHL3* and *BAG6. Patho-scores* between these genes and the “colon” were found to be significant with *P*-value of .019 and .040 respectively (Fig. 5B)*.* Among these, we observed *BAG6* overexpression to be linked with poor overall survival (Fig. 5C). Notably, the first paper connecting UCHL3 with colorectal cancer was published in 2020 (Li *et al.* 2020). While *BAG6* has been linked with an increased risk of lung cancer (Kawahara *et al.* 2013), no noteworthy study was spotted associating the gene with colorectal cancer. We predict, thorough molecular investigation of the gene with a clinical outlook will unleash its mode of action.

## 4 Conclusion

We fine-tuned the BERT base pretrained model using ∼4.5 million abstracts citing disease–gene relationships. The obtained model, called PathoBERT, was subjected to various inference tasks concerning diseases, genes, organs, and cell types. These include inspecting word analogies combining different biological concepts such as disease, genes and symptoms, evaluating the performance of disease–gene association prediction, and discovering new relationships that appeared later in the literature or are still illusive. In all cases PathoBERT showed sufficient promise. In essence, PathoBERT/Pathomap taken together provides a strategy to obtain an unbiased continuous representation of disease-causing genes and their tissue/cell-type specific activities. This can accelerate targeted investigations into diverse diseases. *Patho-scores* and associated color intensities indicate the extent of a gene’s involvement in causing disease in a particular tissue/organ. Pathomap does not provide specifics of a gene’s involvement in a particular disease. To find the mode-of-action (such as mutation, regulation, acetylation, DNA methylation) of a gene in a disease, one can refer to the web server DiGSeE (Kim *et al.* 2017).

A major caveat of the current PathoBERT model is that it is not trained to discriminate among species. As such, the current scores are not resolved at the level of mice, humans, or other primates. We were left with only ∼1.2 million abstracts as we filtered for human related publications, which is not enough for language modeling tasks. We believe this will not introduce any nuisance factors since animal models are typically used to mimic human diseases only.

We did strict benchmarking of different embedding techniques in terms of their ability to infer true associations between diseases and genes, treating manually curated relations as the gold standard. PathoBERT was found to be the leading approach with BioSentVec being at the second spot. The remaining approaches including Word2vec largely failed. While human cell atlasing is a reality, a pathological atlas might take significant effort and resources, which we predict to not arrive anytime soon. Till the time this vision becomes a reality, we trust that Pathomap will assist the community in narrowing the search space of genetic hits. Further, PathBERT can be exploited for deciding genes for diagnostic gene-panels. We demonstrated Pathomap’s ability to synthesize previously unknown associations between diseases and genes scopes. PathoBERT may also serve as an orthogonal approach for Gene Ontologies for the narrow and precise investigation of outputs from high-throughput experiments such as RNA-Seq or MicroArray. It can also assist in prioritizing the gene selection for the large-scale loss of function studies. We provided a template for such analyses by elucidating the potential role of BAG6 colorectal cancer pathogenesis and prognosis.

## Supplementary Material

btae185_Supplementary_Data

## Data Availability

PathoBERT pretrained model: https://github.com/Priyadarshini-Rai/Pathomap-Model. BioSentVec-based abstract classification model: https://github.com/Priyadarshini-Rai/Pathomap-Model. Pathomap R package: https://github.com/Priyadarshini-Rai/Pathomap.
