# Supplementary material for: Literature mining discerns latent disease–gene relationships
Source: Bioinformatics. 2024 Apr 12;40(4):btae185. doi: 10.1093/bioinformatics/btae185 (PMC11060865; doi:10.1093/bioinformatics/btae185)
Supplement: btae185_Supplementary_Data [file btae185_supplementary_data.docx]

## **Supplementary Material**

Literature mining discerns latent disease-gene relationships

Priyadarshini Rai^1^, Atishay Jain^2†^,  Shivani Kumar^2†^, Divya Sharma^1†^, Neha Jha^1^, Smriti Chawla^1^, Abhijit Raj^1^, Apoorva Gupta^3^, Sarita Poonia^1^, Angshul Majumdar^4^, Tanmoy Chakraborty^5,6*^, Gaurav Ahuja^1,7*^ and Debarka Sengupta^1,2,7*^

^1^Department of Computational Biology, Indraprastha Institute of Information Technology-Delhi (IIIT-Delhi), Okhla, Phase III, New Delhi-110020, India; ^2^Department of Computer Science and Engineering, Indraprastha Institute of Information Technology-Delhi (IIIT-Delhi), Okhla, Phase III, New Delhi-110020, India; ^3^Department of Biotechnology, Delhi Technological University, Shahbad Daulatpur, Main Bawana Road, Delhi-110042, India; ^4^IAI, TCG CREST, Kolkata, India; ^5^Department of Electrical Engineering, Indian Institute of Technology Delhi, New Delhi-110016, India; ^6^Yardi School of Artificial Intelligence, Indian Institute of Technology Delhi, New Delhi-110016, India; ^7^Centre for Artificial Intelligence, Indraprastha Institute of Information Technology-Delhi (IIIT-Delhi), Okhla, Phase III, New Delhi-110020, India

^†^ These authors contributed equally.

*To whom correspondence should be addressed.

**Supplementary Table S1:** The below table depicts the performance of different classification algorithms achieved while separating the abstracts featuring disease-gene association and ones that are generic in nature. Finally, the extreme gradient boosting (XGBoost) was used as a classifier for classification.

| **Embedding Type** | **Model** | **Accuracy** | **Precision** | **Recall** | **F1** | **Kappa Score** |
| --- | --- | --- | --- | --- | --- | --- |
| **BioBERT** | SVM | 92.19 | 94.39 | 94.13 | 94.26 | 82.02 |
|  | XGBoost | 92.57 | 93.93 | 95.25 | 94.59 | 82.73 |
|  | LR | 90.85 | 93.53 | 93.01 | 93.27 | 78.99 |
|  | MLP | 92.57 | 96.5 | 92.45 | 94.43 | 83.27 |
|  | Random Forest | 88.95 | 94.91 | 88.54 | 91.61 | 75.47 |
| **Word2vec** | SVM | 88.95 | 93.71 | 89.42 | 91.52 | 75.69 |
|  | XGBoost | 85.33 | 85.63 | 93.71 | 89.49 | 65.36 |
|  | LR | 86.66 | 91.17 | 88.57 | 89.85 | 70.42 |
|  | MLP | 87.23 | 91.98 | 88.57 | 90.24 | 71.8 |
|  | Random Forest | 69.52 | 98.46 | 55.14 | 70.69 | 43.79 |
| **BioSentVec** | SVM | 92.95 | 95.36 | 94 | 94.67 | 84.25 |
|  | XGBoost | **93.14** | 94.6 | 95.14 | **94.87** | **84.52** |
|  | LR | 90.09 | 93.31 | 91.71 | 92.5 | 77.9 |
|  | MLP | 92.57 | 94.55 | 94.28 | 94.44 | 83.3 |
|  | Random Forest | 88.38 | 98.65 | 83.71 | 90.57 | 75.69 |


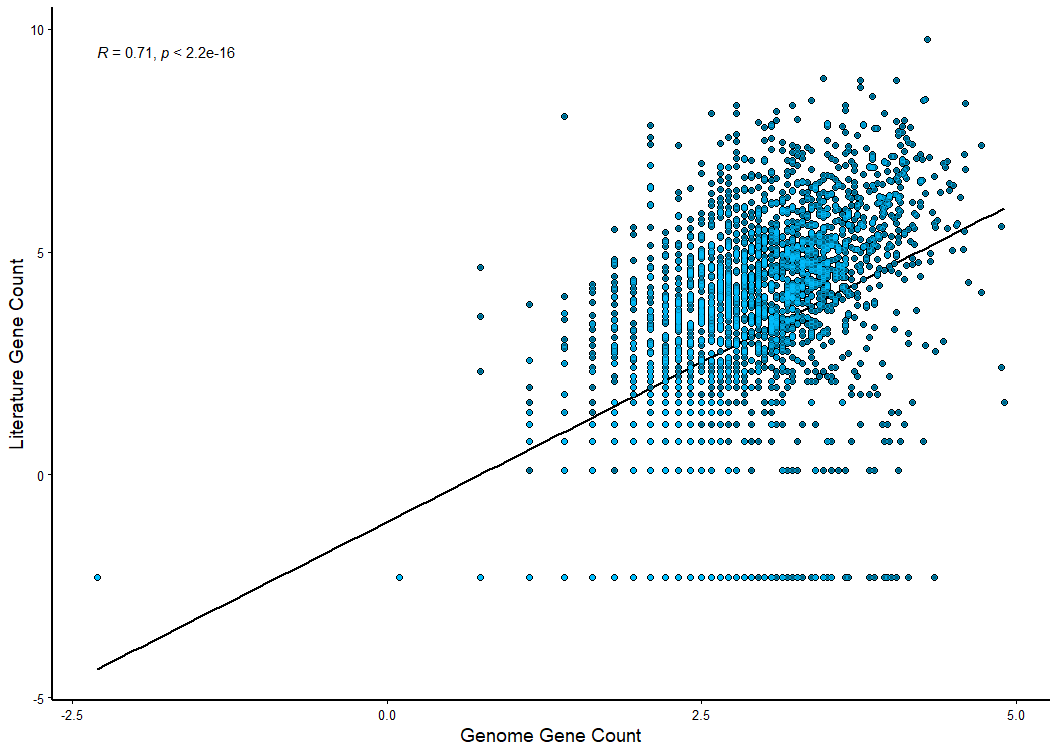


**Supplementary Figure S1.** Correlation between gene numbers across all 1 million bp windows of the human DNA and frequency of their occurrence (genes’) in *patho-abstracts*.


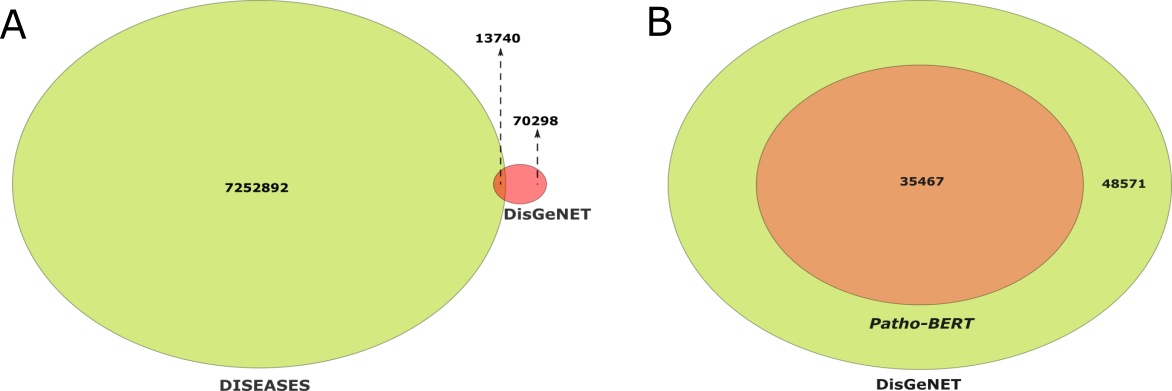


**Supplementary Figure S2.** (A) Intersection of gene-disease pairs between DisGeNET and DISEASES database. Merely ~13,000 of ~84,000 DisGeNET associations detected by DISEASES. (B) PathoBERT showed statistically significant associations for ~35,000 of the DisGeNET pairs. Notably PathoBERT is capable of producing a disease-gene association score for every possible pair. As such, it can be used for discovering novel associations.


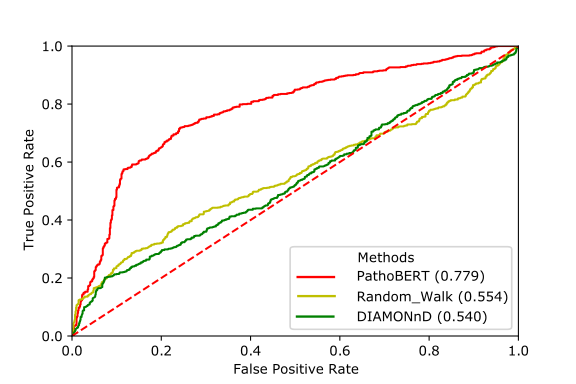


**Supplementary Figure S3.** ROC plots and associated AUC values depicting the accuracy yielded using different methods in recognizing true gene-disease associations as compared to randomly generated ones.


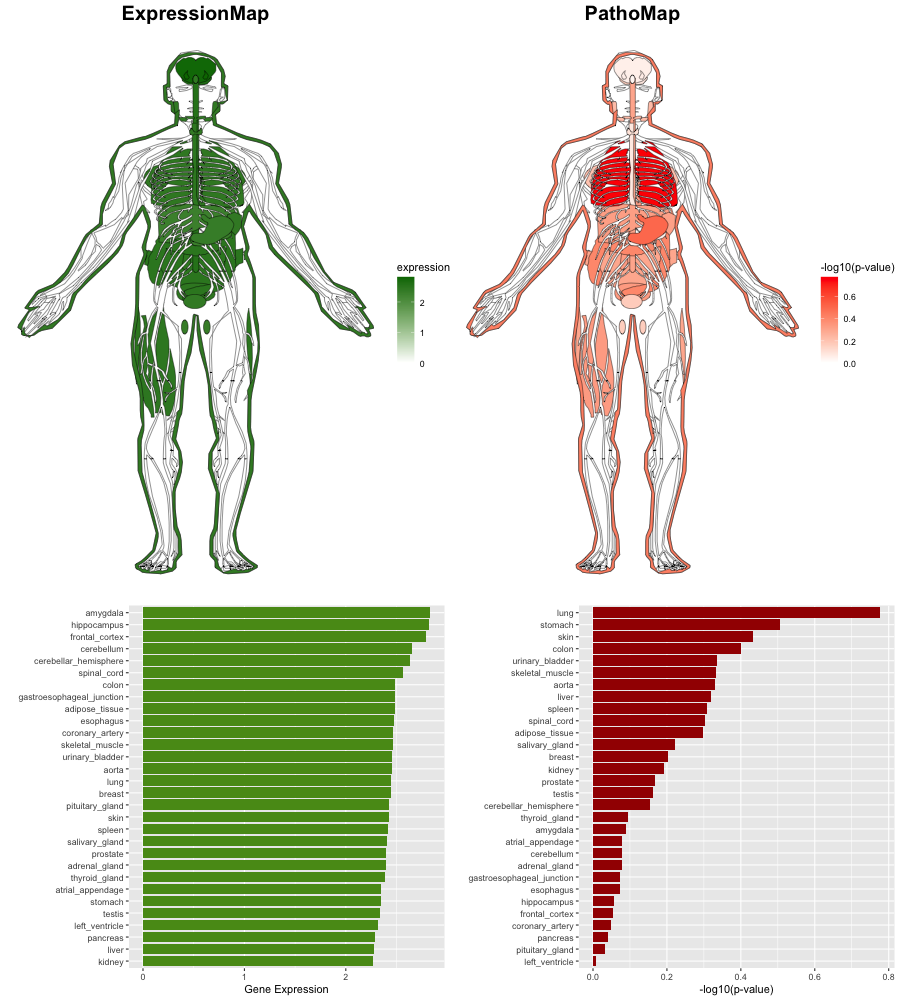


**Supplementary Figure S4.** Distribution of patho-scores and gene expression across organs for APC. The majority of instances of familial adenomatous polyposis (FAP) are caused by germline mutations in the APC gene (Leoz *et al.*, 2015). However, mutations in APC genes are not only limited to FAP but they are also known to play an important role in gastric carcinogenesis (Fang *et al.*, 2002). Furthermore, in colorectal cancers, APC mutations play a rate-limiting role (Fodde 2002).


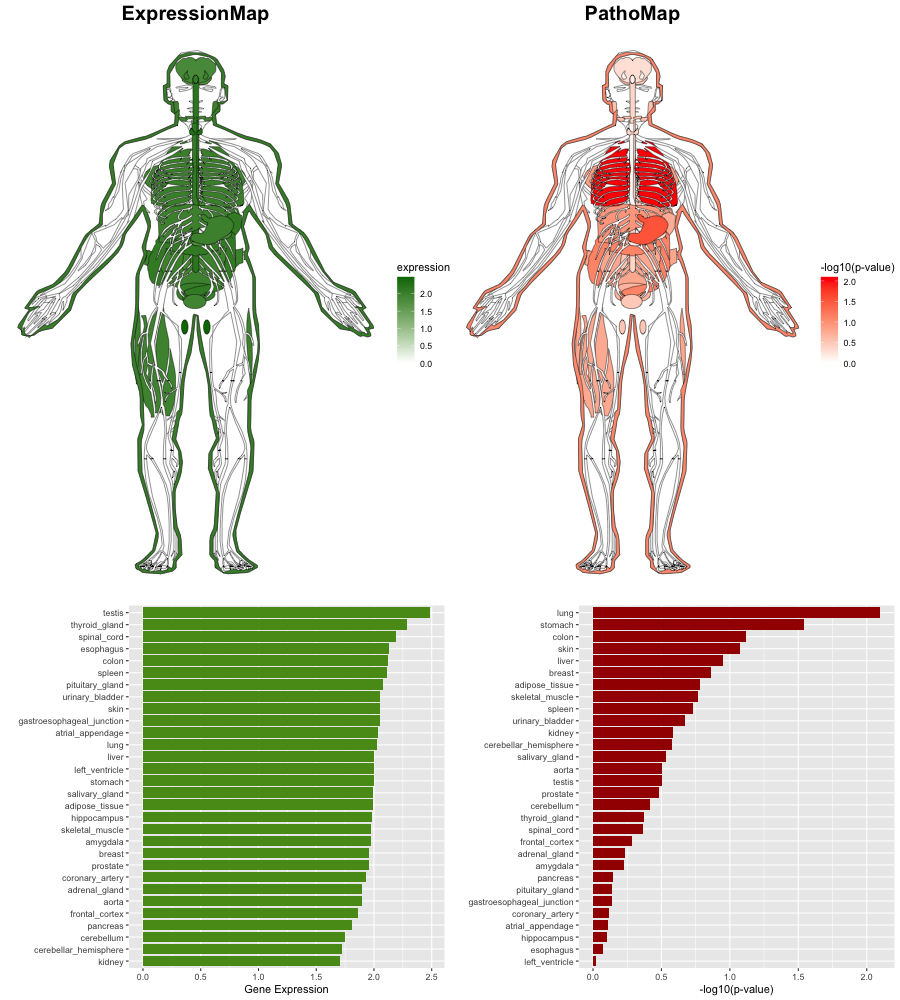


**Supplementary Figure S5.** Distribution of patho-scores and gene expression across organs for BRCA1**.** BRCA1 is a tumor suppressor gene. However, alterations or mutations in this gene predispose individuals to increased risk of certain cancers such as breast, stomach, colorectal cancer, and other cancer types (Godet and Gilkes, 2017) (Levine *et al.*, 2003) (Sopik *et al.*, 2015). Overexpression of BRCA1 is strongly linked to poor survival outcomes in non-small-cell lung cancer (NSCLC) (Rosell *et al.*, 2007).


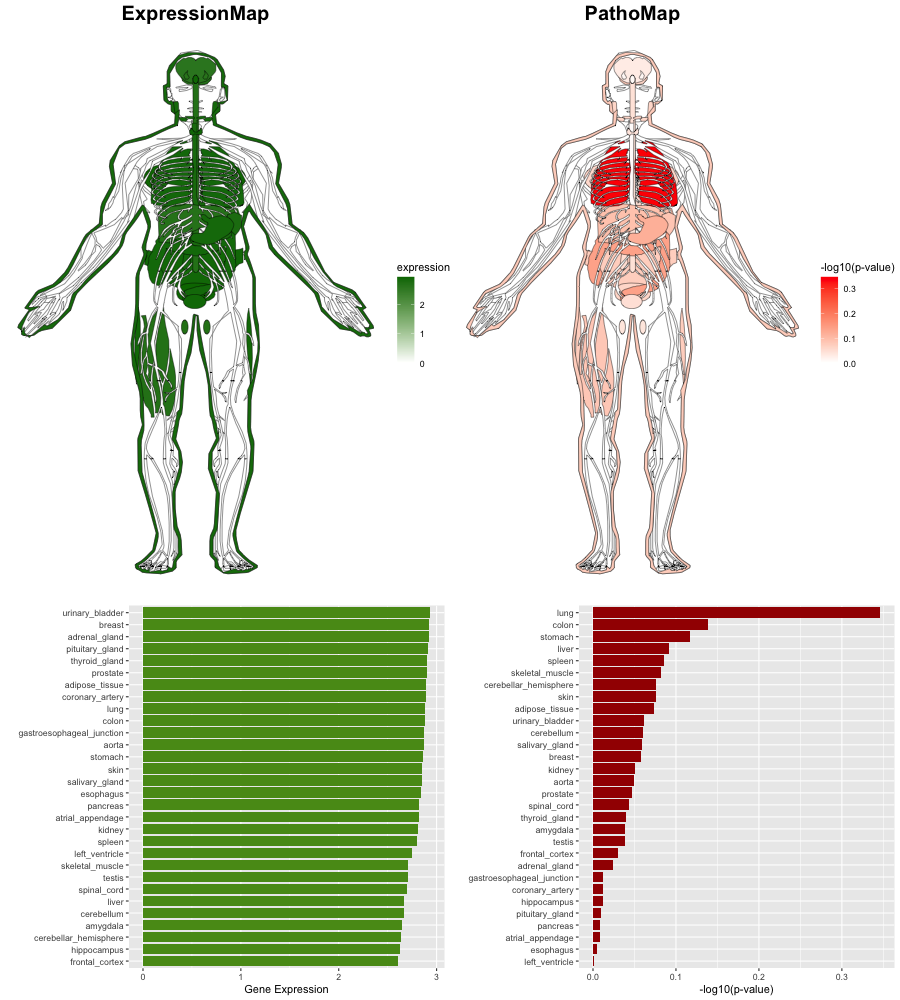


**Supplementary Figure S6.** Distribution of patho-scores and gene expression across organs for CDK4. Cyclin-dependent kinases (CDKs) regulate cell cycle checkpoint and transcriptional events and thus are key regulators of cell proliferation. Notably, dysregulation in CDKs results in uncontrolled cell proliferation. (Ding et al. 2020). Overexpression of CDK4 has been linked with the development and poor prognosis of lung cancer (Wu *et al.*, 2011).


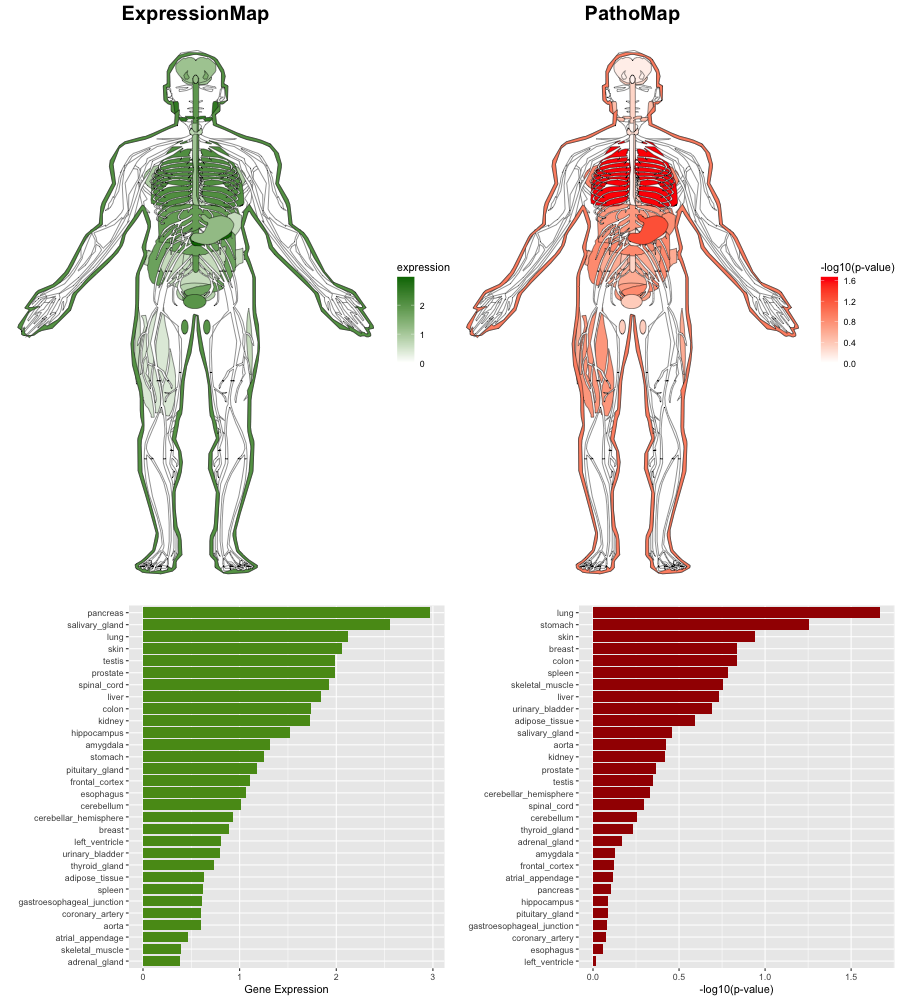


**Supplementary Figure S7.** Distribution of patho-scores and gene expression across organs for CFTR**.**  Cystic fibrosis (CF) is an inherited lethal disorder caused by mutations in the CFTR gene (Mall and Hartl, 2014). CFTR gene encodes for ion channels, dysfunction or alteration in this gene results in the imbalance of ions and fluids in organs such as airways, intestine, etc. (Lopes-Pacheco, 2019). Multiple organs are affected in CF, however, the most serious clinical implications are related to the lungs (Fraser-Pitt and O’Neil, 2015).


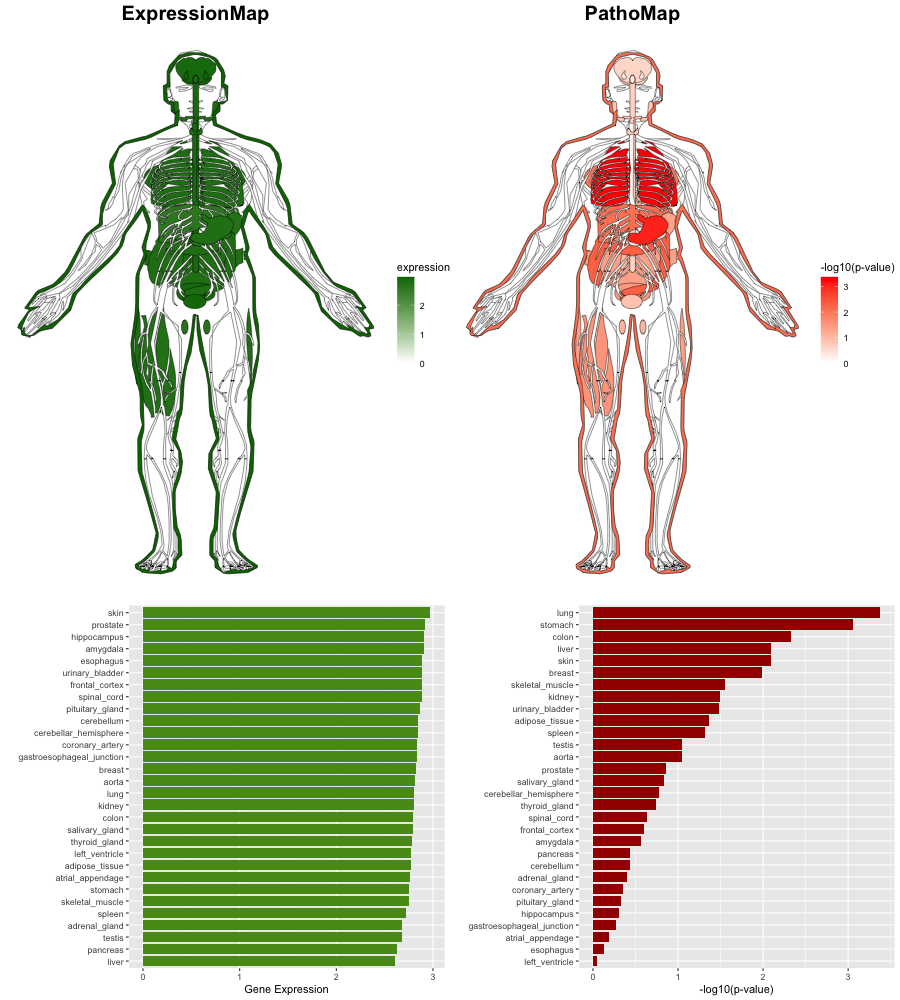


**Supplementary Figure S8.** Distribution of patho-scores and gene expression across organs for HRAS. HRAS is associated with diseases such as Costello Syndrome (Estep *et al.*, 2006) and Epidermal Nevus Syndrome which involves skin-related defects (Kiessling *et al.*, 2015). (Avitan-Hersh *et al.*, 2014)HRAS gene is frequently mutated in lung and bladder cancer and might be a potential therapeutic target (Kiessling *et al.*, 2015). Furthermore, overexpression of HRAS is associated with the progression and poor prognosis of gastric carcinoma (Wu *et al.*, 2016).


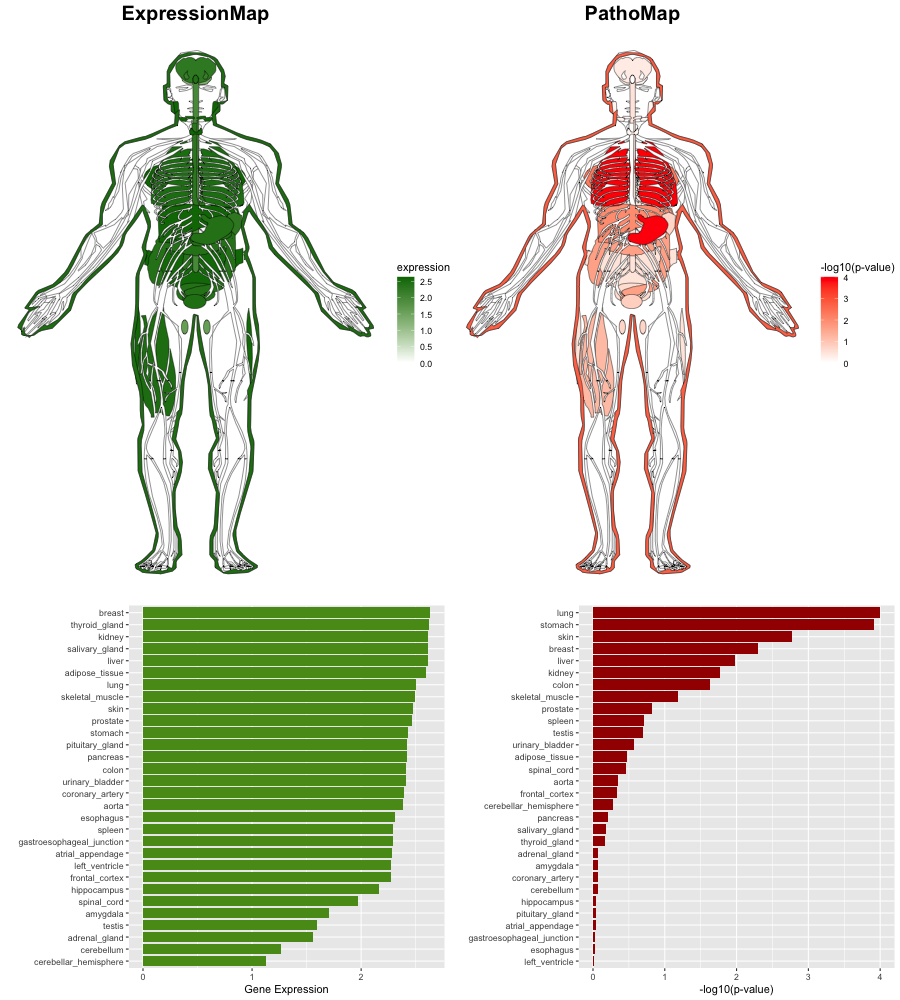


**Supplementary Figure S9.** Distribution of patho-scores and gene expression across organs for MET**.** Mesenchymal Epithelial Transition (MET) is a receptor tyrosine kinase activated by its ligand Hepatocyte Growth Factor (HGF). In many solid cancers, MET is mutated or over-amplified. MET activation appears to be a primary driver of tumorigenesis in lung cancer and might be an effective therapeutic strategy for its treatment (Drilon *et al.*, 2017). Further, overexpression of MET is associated with poor survival outcomes in breast cancer patients (de Melo Gagliato *et al.*, 2014).


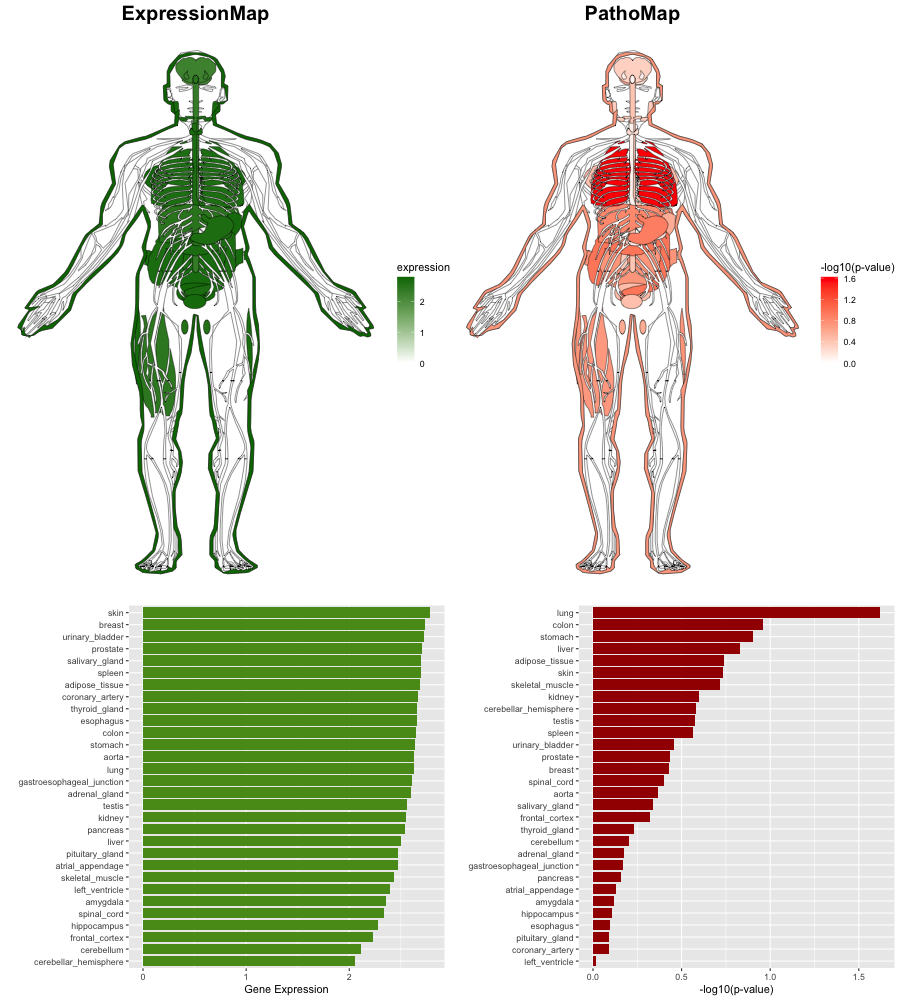


**Supplementary Figure S10.** Distribution of patho-scores and gene expression across organs for TP53**.** TP53 is a tumor suppressor gene that is found to be mutated in a number of cancers including lung cancer. Aberrations in the TP53 gene play an important role in the oncogenesis of lung epithelial cells and are also linked with poor prognosis (Mogi and Kuwano, 2011).

TP53 alterations are also involved in driving the development of colon cancer (Williams *et al.*, 2020).

**References**

Avitan-Hersh,E. *et al.* (2014) Postzygotic HRAS mutation causing both keratinocytic epidermal nevus and thymoma and associated with bone dysplasia and hypophosphatemia due to elevated FGF23. *J. Clin. Endocrinol. Metab.*, **99**, E132–6.

Drilon,A. *et al.* (2017) Targeting MET in Lung Cancer: Will Expectations Finally Be MET? *J. Thorac. Oncol.*, **12**, 15–26.

Estep,A.L. *et al.* (2006) HRAS mutations in Costello syndrome: detection of constitutional activating mutations in codon 12 and 13 and loss of wild-type allele in malignancy. *Am. J. Med. Genet. A*, **140**, 8–16.

Fang,D.-C. *et al.* (2002) Mutation analysis of APC gene in gastric cancer with microsatellite instability. *World J. Gastroenterol.*, **8**, 787–791.

Fraser-Pitt,D. and O’Neil,D. (2015) Cystic fibrosis – a multiorgan protein misfolding disease. *Future Science OA*, **1**.

Godet,I. and Gilkes,D.M. (2017) BRCA1 and BRCA2 mutations and treatment strategies for breast cancer. *Integr. Cancer Sci. Ther.*, **4**.

Kiessling,M.K. *et al.* (2015) Mutant HRAS as novel target for MEK and mTOR inhibitors. *Oncotarget*, **6**, 42183–42196.

Leoz,M.L. *et al.* (2015) The genetic basis of familial adenomatous polyposis and its implications for clinical practice and risk management. *Appl. Clin. Genet.*, **8**, 95–107.

Levine,D.A. *et al.* (2003) Fallopian tube and primary peritoneal carcinomas associated with BRCA mutations. *J. Clin. Oncol.*, **21**, 4222–4227.

Lopes-Pacheco,M. (2019) CFTR Modulators: The Changing Face of Cystic Fibrosis in the Era of Precision Medicine. *Front. Pharmacol.*, **10**, 1662.

Mall,M.A. and Hartl,D. (2014) CFTR: cystic fibrosis and beyond. *Eur. Respir. J.*, **44**, 1042–1054.

de Melo Gagliato,D. *et al.* (2014) Analysis of MET genetic aberrations in patients with breast cancer at MD Anderson Phase I unit. *Clin. Breast Cancer*, **14**, 468–474.

Mogi,A. and Kuwano,H. (2011) TP53 mutations in nonsmall cell lung cancer. *J. Biomed. Biotechnol.*, **2011**, 583929.

Rosell,R. *et al.* (2007) BRCA1: a novel prognostic factor in resected non-small-cell lung cancer. *PLoS One*, **2**, e1129.

Sopik,V. *et al.* (2015) BRCA1 and BRCA2 mutations and the risk for colorectal cancer. *Clin. Genet.*, **87**, 411–418.

Williams,D.S. *et al.* (2020) Overexpression of TP53 protein is associated with the lack of adjuvant chemotherapy benefit in patients with stage III colorectal cancer. *Mod. Pathol.*, **33**, 483–495.

Wu,A. *et al.* (2011) Elevated expression of CDK4 in lung cancer. *J. Transl. Med.*, **9**, 38.

Wu,X.Y. *et al.* (2016) Identification of HRAS as cancer-promoting gene in gastric carcinoma cell aggressiveness. *Am. J. Cancer Res.*, **6**, 1935–1948.
